# Chikungunya virus in Thailand (2020–2023): Epidemiology, clinical features, and genomic insights

**DOI:** 10.1371/journal.pntd.0013548

**Published:** 2025-09-15

**Authors:** Sarawut Khongwichit, Watchaporn Chuchaona, Sumeth Korkong, Lakkhana Wongsrisang, Thanunrat Thongmee, Yong Poovorawan

**Affiliations:** Center of Excellence in Clinical Virology, Department of Pediatrics, Faculty of Medicine, Chulalongkorn University, Bangkok, Thailand; University of Virginia School of Medicine, UNITED STATES OF AMERICA

## Abstract

Chikungunya virus (CHIKV) caused significant outbreaks in Thailand during 2008–2009 and 2018–2020. Despite the COVID-19 pandemic, CHIKV continued to circulate; however, data on its epidemiological, clinical, and genetic characteristics during and after this period remains limited. This study investigated CHIKV infections in Thailand from March 2020 to December 2023. Serum samples (n = 1,264) were collected from patients with suspected CHIKV infection at 14 hospitals across five provinces in central, eastern, and northeastern Thailand. Samples were tested by RT-qPCR and IgM fluorescence immunoassay. CHIKV infection was confirmed in 50.5% (638/1,264) of cases. Infections occurred across all age groups, with the highest prevalence among individuals aged ≥56 years. Clinical symptoms significantly associated with infection included myalgia, arthralgia, rash, and conjunctivitis. Rash was more frequently in individuals aged ≤15 years and was significantly associated with lower viral loads. Arthralgia was more common among older adults and was linked to later illness onset. Myalgia was least frequently reported in younger patients. Thirty-eight complete coding sequences of our Thai CHIKV strains were analyzed in phylogenetic and time-scaled trees alongside 186 global strains and 109 ECSA-IOL strains from GenBank, respectively. Genome analysis revealed that CHIKV strains circulating in Thailand during 2020–2023 belonged to the East/Central/South African–Indian Ocean lineage (ECSA-IOL). These strains did not evolve from earlier ECSA-IOL variants that carried the E1-A226V mutation, which was previously detected in Thailand. Instead, all isolates carried E1-K211E and E2-V264A, along with E1-226A, likely introduced from the Indian subcontinent around 2016–2017. This introduction triggered a major outbreak between late 2018 and 2020, followed by sustained transmission. The 2020–2023 Thai strains exhibited high genetic similarity to those from neighboring countries, with multiple nonsynonymous mutations suggesting ongoing viral adaptation. Understanding CHIKV epidemiology, clinical features, and evolution supports improved surveillance, diagnostics, and public health interventions.

## Author summary

This study examined the prevalence and clinical features of chikungunya virus infections and described the genetic evolution of the virus from 2020 to 2023. We found that CHIKV affected individuals across all age groups, with a higher incidence among those aged ≥56 years. Symptoms varied by age, with older adults predominantly experiencing joint pain and younger children more likely to develop skin rashes. Furthermore, we observed that viral load in infected patients correlated with symptoms such as fever, headache, and rash. Genomic analysis revealed that the virus strains circulating during this period belonged to the Indian Ocean Lineage of the East/Central/South African genotype. These strains carry mutations that enhance the fitness of *Aedes aegypti* mosquitoes and were probably introduced into Thailand from the Indian subcontinent around 2016–2017, leading to a major outbreak from 2018 to 2020 and continuing circulation to the present. Our study also found that the CHIKV Thai strains from 2020 to 2023 were genetically similar to those associated with recent outbreaks in neighboring countries, suggesting ongoing regional transmission. We believe that our findings may inform enhanced surveillance, improve diagnostic approaches, and guide targeted public health interventions to mitigate the spread of CHIKV.

## Introduction

Chikungunya virus (CHIKV) is a mosquito-borne alphavirus in the family *Togaviridae*, primarily transmitted by *Aedes aegypti* and *Aedes albopictus* mosquitoes [1]. CHIKV is the etiological agent of an acute febrile illness that can present with a broad spectrum of clinical manifestations that often overlap with those of dengue and Zika virus infections, leading to diagnostic challenges [2]. Although CHIKV infection is rarely fatal, it can result in chronic and debilitating joint symptoms [3]. Atypical manifestations and life-threatening complications affecting the cardiovascular, neurological, renal, ocular, and other organ systems have also been documented [4–8]. Despite the approval of a vaccine, no specific antiviral therapy is currently available [9]. CHIKV is phylogenetically classified into three principal genotypes: West African, Asian, and East/Central/South African (ECSA) [10]. Within the ECSA genotype, the Indian Ocean lineage (ECSA-IOL) emerged in Kenya in 2004 as a distinct epidemic clade, subsequently spreading to the Indian Ocean islands, the Indian subcontinent, Asia, and Europe [11,12]. In late 2013, the first documented local transmission of chikungunya virus (CHIKV) occurred in the Caribbean islands, involving the Asian genotype. The virus rapidly spread across the Americas [13,14], with subsequent outbreaks involving both the Asian and ECSA genotypes, depending on region and period. These genotypes were associated with distinct regional lineages: the Asian-American lineage, which predominated in early outbreaks in the Caribbean and Central America, and the ECSA-American lineage, a distinct clade from the ECSA-IOL, which was responsible for later outbreaks in South America [15]. Since then, CHIKV has continued to circulate globally, with reported cases now spanning more than 100 countries across Africa, Asia, Europe, and the Americas [16].

In Thailand, CHIKV has circulated since the 1950s, with the first recorded case reported in Bangkok in 1958 [17]. Between 1976 and 1995, only sporadic cases were documented across various provinces, with all strains identified during that period belonging to the Asian genotype [18]. The largest reported outbreak of CHIKV in Thailand occurred in 2008–2009, primarily affecting southern provinces, with more than 50,000 cases reported [19,20]. A smaller, localized outbreak followed in 2013 in Bueng Kan province, northeastern Thailand [21]. Since then, the incidence of CHIKV in Thailand had remained relatively low until 2018. In mid-2018, however, case numbers began to increase, resulting in a nationwide resurgence, with approximately 28,000 confirmed cases reported between 2018 and 2020 [19]. Phylogenetic analyses revealed that the virus responsible for the significant outbreaks belonged to the ECSA-IOL lineage and harbored key distinguishing mutations [22]. Between 2020 and 2023, the incidence of CHIKV in Thailand showed notable fluctuations, influenced by the concurrent COVID-19 pandemic and the transition to the post-pandemic phase. According to the Bureau of Epidemiology, Ministry of Public Health of Thailand [19], chikungunya reported incidence rates declined from 17.07 cases per 100,000 population in 2020 to 1.01 in 2021. However, incidence rates gradually increased thereafter, rising to 1.98 and 2.25 cases per 100,000 in 2022 and 2023, respectively.

Although CHIKV has been studied in Thailand for several decades, most previous research has examined epidemiological trends, clinical manifestations, and viral genetics in isolation. Integrated studies combining these aspects are still limited and often constrained by small sample sizes, outdated genomic data, and restricted geographic coverage. Notably, no comprehensive investigation has concurrently assessed the epidemiology, clinical characteristics, and genomic features of CHIKV infections, particularly during and after the COVID-19 pandemic. Accordingly, this study aimed to evaluate the prevalence and clinical features of CHIKV infections, assess associations between viral load and symptoms, and characterize the genetic diversity and evolution of circulating strains in Thailand from March 2020 to December 2023.

## Materials and methods

### Ethics statement

This study was approved by the Institutional Review Board of the Faculty of Medicine, Chulalongkorn University, Thailand (approval no. IRB710/64). The requirement for informed consent was waived by the Ethics Committee for Human Research, as all patient identifiers were anonymized before data analysis to ensure confidentiality and compliance with the Declaration of Helsinki.

### Study samples

A total of 1,264 serum samples were collected from patients with suspected CHIKV infection who visited hospitals between March 2020 and December 2023. Suspected cases were defined as patients presenting with acute fever (≥38.5°C), with or without arthralgia, particularly those residing in, or having a travel history to, areas with known CHIKV transmission globally. Travel history was reported by patients and recorded by hospital staff at the time of sample collection. Serum samples were obtained from 14 collaborating hospitals located in five provinces across three regions of Thailand: Central (Bangkok, Samut Prakan, Samut Sakhon), Eastern (Chon Buri), and Northeastern (Surin).

Inclusion in the study was determined by attending physicians at participating hospitals, based on the suspected CHIKV case definition. Recruitment required submission of diagnostic specimens and case record forms (CRFs) by these physicians. The CRFs recorded patient demographic information (date of birth, age, sex), clinical presentation (illness onset date, symptoms), and travel history. The serum samples and corresponding CRFs were transported to the Center of Excellence in Clinical Virology at King Chulalongkorn Memorial Hospital, Bangkok, Thailand, for laboratory testing and clinical data analysis. Information on whether patients were inpatients or outpatients was not recorded in our dataset. Exclusion criteria included cases with incomplete CRF data required for clinical and statistical analyses, and cases with confirmed CHIKV infection that showed co-infection with dengue virus (DENV) or Zika virus (ZIKV). The timing of symptom onset was not part of the inclusion criteria, but it was recorded at enrolment based on patient or caregiver reports. All samples were tested for CHIKV, DENV, and ZIKV by RT-PCR, as described. A confirmed CHIKV infection was defined as the detection of CHIKV-specific IgM antibodies or viral RNA. Patients with detectable CHIKV-specific IgG antibodies but negative for IgM were classified as having a prior CHIKV infection. The overall workflow for patient enrollment, laboratory testing, and selection of samples for viral load quantification and phylogenetic analyses is summarized in S1 Fig.

### RNA extraction and CHIKV detection using quantitative reverse transcriptase polymerase chain reaction (RT-qPCR) amplification

RNA was extracted from 200 μL of serum using the MagDEA Dx SV kit (Precision System Science, Chiba, Japan) with the automated magLEAD 12gC instrument according to the manufacturer’s instructions. CHIKV RNA was detected using a qRT-PCR assay targeting a conserved region of the nonstructural protein 4 (nsP4) gene. Primer and probe sequences were used as described by Lanciotti et al. [23], including CHIKV_6856Fw (5′-TCACTCCCTGTTGGACTTGATAGA-3′), CHIKV_6981Rv (5′-TTGACGAACAGAGTTAGGAACATACC-3′), and the CHIKV-FAM probe (5′-AGGTACGCGCTTCAAGTTCGGCG-3′). The 20 μL reaction volume contained 5 μL of 4X TaqMan Fast Virus 1-Step Master Mix (Thermo Fisher Scientific, USA), 0.25 μM of each primer, 0.125 μM of probe, 5 μL of extracted RNA, and nuclease-free water. Amplification was performed on an ABI ViiA 7 Real-Time PCR System (Thermo Fisher Scientific, Wilmington, DE) using the following cycling conditions: 50°C for 10 minutes (reverse transcription), 95°C for 20 seconds (initial denaturation), followed by 45 cycles of 95°C for 3 seconds and 60°C for 30 seconds. A sample was considered positive if the cycle threshold (Ct) value was ≤ 38. Each run included a no-template control, a CHIKV-positive control derived from RNA extracted from cultured virus stock, and an internal amplification control targeting the human RNase P gene to assess sample quality and extraction efficiency. All serum samples were also tested for dengue virus (DENV) and Zika virus (ZIKV) RNA using virus-specific real-time RT-PCR assays, as previously described [24,25].

### Detection of anti-CHIKV IgM and IgG antibodies

Anti-CHIKV IgM and IgG antibodies were detected using the STANDARD F Chikungunya IgM/IgG fluorescence immunoassay (FIA) kit (SD BIOSENSOR, Gyeonggi-do, Korea), following the manufacturer’s instructions. Briefly, 10 μL of undiluted serum was applied to the FIA cassette and incubated at room temperature for 15–20 minutes. Results were interpreted using the STANDARD F200 Analyzer (SD BIOSENSOR, Gyeonggi-do, Korea) based on fluorescence intensity.

### Quantification of CHIKV viral load

The primers and probe targeting the nsP4 gene were adopted from a previously validated qRT-PCR assay described by Lanciotti et al. [23]. This assay targets the relatively conserved nsP4 region and has been widely used in CHIKV molecular diagnostics and viral load quantification due to its high analytical sensitivity and specificity [26–30]. In our study, the same primer/probe set was used for CHIKV RNA detection; therefore, we applied the same target region for quantification to ensure consistency across both diagnostic and quantitative analyses. Viral load quantification was conducted on RNA-positive samples using qRT-PCR with a standard curve generated from in vitro-transcribed CHIKV RNA. A fragment of the nsP4 gene encompassing the primer/probe binding sites was cloned into the T&A Cloning Vector (Yeastern Biotech, Taipei, Taiwan) and subsequently transformed into *Escherichia coli* DH5α-competent cells. Recombinant plasmids were purified utilizing the Exprep Plasmid SV Kit (GeneAll Biotechnology, Seoul, Korea) and employed as templates for in vitro transcription using the MEGAscript T7 Transcription Kit (Invitrogen, Carlsbad, CA), according to the manufacturer’s instructions. The transcribed RNA was purified, and its concentration was determined using a NanoDrop Spectrophotometer (Thermo Fisher Scientific, Waltham, MA). RNA copy numbers were calculated, and a tenfold serial dilution series (10^1^–10^10^) was prepared to construct the standard curve. The assay yielded a slope of –3.402, a Y-intercept of 39.83, a coefficient of determination (R^2^) of 0.997, and an amplification efficiency of 96.77%. The Ct values obtained for each patient sample were compared against the standard curve to estimate the viral load in serum samples. The viral load was expressed as RNA copies per milliliter (copies/mL) of serum. Standard curve parameters are shown in S2 Fig.

### Genome sequencing and phylogenetic analysis

The complete coding sequences of CHIKV were amplified using the SuperScript IV One-Step RT-PCR System (Invitrogen, Carlsbad, CA) with specific primers listed in S1 Table [31]. PCR products were purified using the GeneAll Gel Extraction Kit (GeneAll Biotechnology, Seoul, Korea) and sent for next-generation sequencing at Tsingke Biotechnology Co., Ltd. (Hangzhou, China). Library preparation was conducted using the S-TN5 transposase-based method (TDE0506), and sequencing was performed on the Illumina MiSeq platform (MiSeq Reagent Micro Kit V2), generating 150 bp paired-end reads. Raw reads underwent quality control using fastp (v0.20.1) [32] to trim adapters and filter low-quality sequences. High-quality reads were assembled using SPAdes [33], followed by reference-based mapping to the CHIKV prototype strain S27 (GenBank accession no. AF369024).

After receiving consensus sequences from the sequencing provider, we used CHROMAS LITE v2.6.6 (http://www.technelysium.com.au/chromas.html; Technelysium Pty Ltd, Australia) to manually review base quality and sequencing peaks. BLAST analysis (https://blast.ncbi.nlm.nih.gov/Blast.cgi) was performed on the assembled sequences to confirm CHIKV identity, assess genome completeness, and annotate gene regions. Final assemblies of the complete coding region (11,238 nt) were generated using BioEdit v7.7.1 [34]. The nucleotide sequences generated in this study have been deposited in GenBank under accession numbers PQ637673–PQ637710.

A total of 38 CHIKV-positive samples were selected for sequencing based on criteria including low cycle threshold (Ct) values (<30), representation across different geographic sites, and coverage of multiple months and years to ensure both genetic quality and spatiotemporal diversity (S1 Fig). For maximum-likelihood phylogenetic analysis, the complete coding sequences of the 38 CHIKV isolates generated in this study (collected between 2020 and 2023) were aligned with 186 publicly available CHIKV sequences from GenBank, representing multiple genotypes and geographic regions, to assess genotype clustering and global diversity. Multiple sequence alignments were performed using MUSCLE [35] under default settings, as implemented in MEGA11 [36]. A maximum-likelihood (ML) phylogenetic tree was constructed using IQ-TREE v2 [37] with 1,000 ultrafast bootstrap replicates [38] and the best-fit nucleotide substitution model (GTR + F + I + G4), selected using ModelFinder based on the Bayesian Information Criterion (BIC) [39]. The resulting phylogenetic tree was midpoint rooted and visualized using the Interactive Tree of Life (iTOL) v7 (https://itol.embl.de/) [40].

### Pairwise nucleotide identity analysis

Pairwise nucleotide identity was assessed based on the complete coding sequences of CHIKV isolates. Sequences were aligned using MUSCLE prior to distance estimation. Nucleotide similarity among CHIKV sequences was calculated in MEGA11 using the Compute Pairwise Distances function under the Maximum Composite Likelihood model. All codon positions (1^st^–3^rd^) and noncoding sites were included in the analysis; gaps were treated by complete deletion, and a bootstrap analysis with 1,000 replicates was performed.

### Time-scaled phylogenetic analysis of East/Central/South African genotype–Indian Ocean lineage (ECSA-IOL) CHIKV

To assess the evolutionary dynamics of the CHIKV ECSA-IOL lineage, we applied a Bayesian phylogenetic framework to a dataset comprising 147 complete coding sequences, including isolates from both historical outbreaks and recent epidemics. This dataset included 38 sequences generated in the present study (2020–2023) and 109 publicly available sequences retrieved from GenBank. Collectively, these sequences represent a broad temporal and geographic range of ECSA-IOL viruses sampled between 2004 and 2023. Before Bayesian analysis, the dataset’s suitability for time-scaled phylogenetic inference was evaluated by assessing the temporal signal using root-to-tip regression analysis in TempEst v1.5.3 [41]. The analysis demonstrated a strong clock-like structure (R^2^ = 0.967; correlation coefficient = 0.983), confirming that the dataset was appropriate for molecular clock modeling. Bayesian time-scaled phylogenies were reconstructed using Markov chain Monte Carlo (MCMC) sampling implemented in BEAST v1.10.4 [42]. The analyses employed the SRD06 nucleotide substitution model, an uncorrelated lognormal relaxed molecular clock, and the Bayesian skyline coalescent tree prior [43] to estimate the time to the most recent common ancestor (TMRCA) and evolutionary rates. Bayesian MCMC analyses were performed for a total of 600 million generations, comprising four independent replicates of 150 million generations each, with sampling every 10,000 generations. The XML input files (S1 File) and MCMC log outputs (S2–S3 Files) from the four independent BEAST runs are provided. Independent runs were combined using LogCombiner v1.10.4 after discarding the first 10% of trees as burn-in. Convergence of parameters and effective sample sizes (ESS > 200) were verified using TRACER v1.7.2 [44]. The maximum clade credibility (MCC) tree was generated with TreeAnnotator v1.10.4 and visualized using FigTree v1.4.4 [45].

### Data analysis

Categorical variables, including patient sex, age group, and clinical features, were summarized as frequencies and percentages (n [%]). Patient age and time to disease onset detection were reported as medians with interquartile ranges (IQRs). Chi-square tests and logistic regression analyses were conducted to evaluate demographic characteristics and the occurrence of symptoms across groups. Correlations between clinical features and categorical variables, such as age group, were assessed using the Pearson chi-square test or Fisher’s exact test, as appropriate. CHIKV log₁₀ viral load was compared between groups defined by binary symptom status and across illness days using t-tests. Annual chikungunya case counts from the Ministry of Public Health (MoPH), Thailand [19], were summarized descriptively and visualized as annual bar charts and monthly heat maps using GraphPad Prism version 10.4.2 (GraphPad Software, San Diego, CA). Multivariable logistic regression was used to evaluate independent associations between key symptoms (fever, rash, arthralgia, myalgia, conjunctivitis) and covariates (age, sex, day since illness onset, and log₁₀ CHIKV viral load). All statistical analyses were performed using SPSS Statistics version 28 (IBM Corporation, Armonk, NY, USA). *p*-values <0.05 were considered statistically significant. For analyses involving multiple comparisons, *p*-values were adjusted using the false discovery rate (FDR) method, with FDR < 0.05 considered statistically significant.

## Results

### Trends in chikungunya virus incidence in Thailand

**Fig 1:**
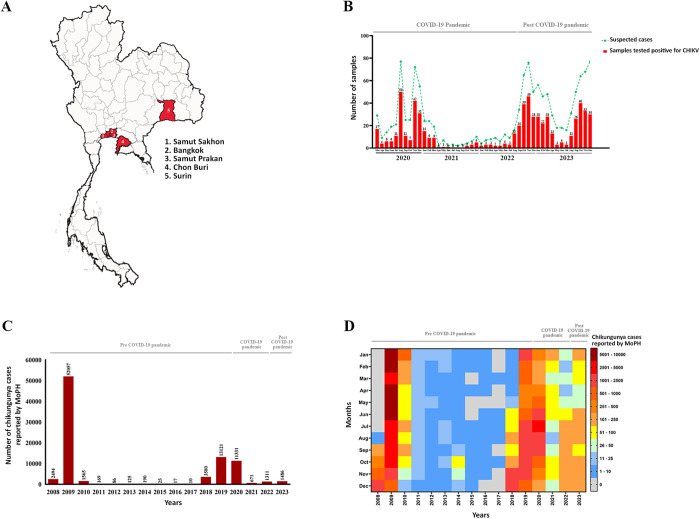
Cohort-based and national surveillance analysis of CHIKV infection in Thailand. (A) Map of Thailand indicating the five locations where samples were collected. Samples were obtained from individuals admitted to hospitals between 2020 and 2023 across five provinces: Bangkok (N = 841), Samut Prakan (N = 352), Samut Sakhon (N = 44), Chon Buri (Eastern region; N = 24), and Surin (Northeastern region; N = 3). The base map was adapted from Northern Thailand map 02.svg by Douglas Paul Perkins, Wikimedia Commons, licensed under CC BY 4.0 (https://commons.wikimedia.org/wiki/File:Northern_Thailand_map_02.svg) and modified by the authors. (B) Monthly counts of suspected and laboratory-confirmed CHIKV cases among study participants from March 2020 to December 2023. Suspected cases are represented by a line graph, while laboratory-confirmed cases are displayed as bar graphs, with exact counts shown above each bar. (C) Annual reported chikungunya cases and (D) heat map displaying the monthly distribution of reported cases from 2008 to 2023, both based on data from the Ministry of Public Health (MoPH), Thailand.

In this study, 1,264 serum samples were collected from individuals with suspected CHIKV infection between March 2020 and December 2023, predominantly from Bangkok and neighboring provinces, with additional samples from Surin province in northeastern Thailand (Fig 1A). The proportion of CHIKV-positive samples was 53.5% (185/346) in 2020, 45.5% (46/101) in 2021, 55.7% (165/269) in 2022, and 46.4% (242/521) in 2023 (Fig 1B). Suspected and confirmed cases were lowest in 2021 and increased beginning in late 2022, consistent with national trends.

At the national level, surveillance data (Fig 1C, D) showed the highest number of chikungunya virus (CHIKV) cases in 2008–2009 (>50,000 cases), followed by a substantial decline during 2010–2017, and a resurgence beginning in mid-2018 that peaked at 13,121 cases in 2019. Incidence remained high in 2020 (11,331 cases), declined sharply in 2021 (671 cases), coinciding with stringent COVID-19 restrictions, and increased again in 2022 (1,311 cases) and 2023 (1,486 cases) following the lifting of control measures.

### Laboratory-confirmed CHIKV infection

**Fig 2:**
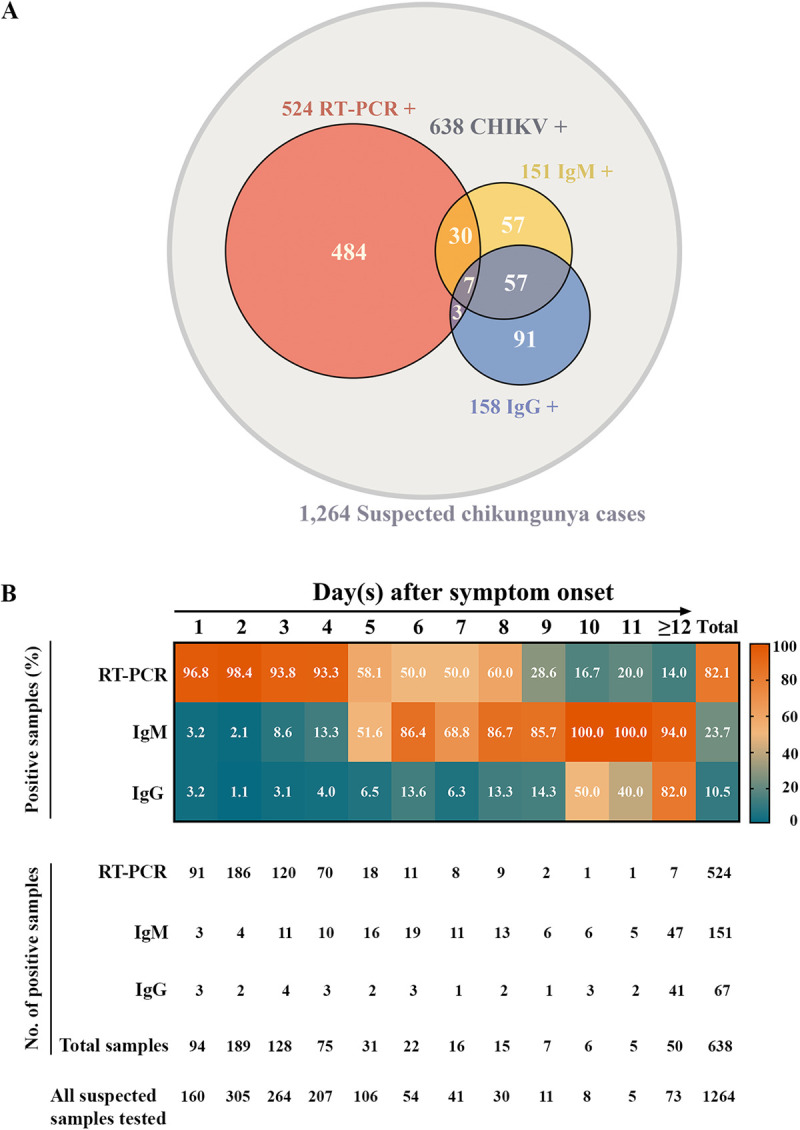
Detection of CHIKV RNA and CHIKV-specific IgM/IgG antibodies. (A) Venn diagram depicting diagnostic results for samples that tested positive by real-time RT-PCR (coral pink circle), IgM fluorescence immunoassay (FIA) (yellow circle), or IgG FIA (blue circle). CHIKV infection was confirmed in 638 individuals based on positive results from real-time RT-PCR and/or CHIKV-specific IgM antibody detection. (B) Positive cases detected by real-time RT-PCR and CHIKV-specific IgM/IgG FIA were categorized by days post-symptom onset. The heatmap displays the percentage of positive samples per assay per day after symptom onset. Below the heatmap, the corresponding number of positive samples and the total number of CHIKV-positive samples collected each day after symptom onset are shown. The bottom row, labeled “All suspected samples tested,” indicates the number of suspected CHIKV cases from which samples were collected each day after symptom onset.

In this study, 1,264 serum samples from patients with suspected CHIKV infection were analyzed by RT-PCR and fluorescence immunoassay (FIA) to detect CHIKV RNA and CHIKV-specific IgM and IgG antibodies (Fig 2A). Of the tested samples, 50.5% (638/1,264) were confirmed positive by RT-PCR and/or IgM FIA. None of the patients reported travel outside their province or to another country within the three weeks prior to symptom onset. Therefore, all confirmed CHIKV cases were classified as autochthonous. Among these, 23.7% (151/638) tested positive for anti-CHIKV IgM antibodies, while CHIKV RNA was detected in 82.1% (524/638). Conversely, 626 samples tested negative for both assays. No cases of co-infection between CHIKV (detected by RT-PCR or IgM) and either DENV or ZIKV (detected by RT-PCR) were observed. Among CHIKV-negative cases (n = 626), 46 were positive for DENV RNA and 71 for ZIKV RNA. Anti-CHIKV IgG antibodies were detected in 12.5% (158/1,264) of the samples, but 7.2% (91/1,264) of individuals showed evidence of past infection based solely on IgG positivity.

Detection efficiencies of RT-PCR and IgM/IgG FIA were assessed relative to symptom onset, as summarized in Fig 2B. During the early phase (days 1–4), RT-PCR demonstrated high sensitivity, with positivity rates exceeding 90%, whereas IgM detection rates were low, at 3.2%, 2.1%, 8.6%, and 13.3% on days 1, 2, 3, and 4, respectively. From day 5, IgM positivity rose markedly, reaching 51.6%. From day 6 onward, IgM sensitivity surpassed that of RT-PCR, with positivity exceeding 90% by day 10. Anti-CHIKV IgG antibodies were rarely detected during days 1–9 but increased sharply thereafter, reaching 50.0% on day 10 and 82.0% by day 12.

### Demographic and clinical characteristics

Among the 1,264 study participants (Table 1), 594 (47.0%) were male and 670 (53.0%) were female, with a median age of 36 years (IQR 25–49; range 3 months–90 years). CHIKV infection was confirmed in 638 cases, comprising 296 (46.4%) males and 342 (53.6%) females. There was no significant difference in infection distribution between sexes (p = 0.667). The median age of confirmed cases was 39 years (IQR 26–53; range 3 months–88 years). Age-specific analysis revealed significant variation in infection rates across age groups (*p* < 0.001), with the highest prevalence among individuals aged ≥ 56 years (62.1%), followed by those aged 46–55 years (57.0%). The lowest prevalence was observed among individuals aged 26–35 years (39.6%). The median interval from illness onset to diagnosis was 3 days (IQR 2–4).

**Table 1:** Demographic and clinical characteristics of individuals according to CHIKV infection status (N = 1,264). The table summarizes the distribution of sex, age, and clinical symptoms among confirmed CHIKV and non-CHIKV cases, along with odds ratios (OR), confidence intervals (CI), and both unadjusted and FDR-adjusted *p*-values for group comparisons.

Characteristics	Suspected cases (N = 1264)	Confirmed cases (N = 638)	Non-CHIKV (N = 626)	OR (95% Cl)	*p*-value	FDR-adjusted *p-value*
**Sex**‒**no./total no. (%)**						
Male	594/1264 (47.0)	296/638 (46.4)	298/626 (47.6)	Reference	0.667	‒
Female	670/1264 (53.0)	342/638 (53.6)	328/626 (52.4)	1.05 (0.84‒1.31)		
**Age**						
Median (interquartile range)	36 yr (25 yr‒49 yr)	39 yr (26 yr‒53 yr)	33 yr (25 yr-47 yr)			
Range	3 mo-90 yr	3 mo‒88 yr	1 yr‒90 yr			
Distribution — no. (%)						
≤15 yr	166 (13.1)	84 (50.6)	82 (49.4)	1.20 (0.78‒1.86)	< 0.001	‒
16-25 yr	161 (12.7)	74 (46.0)	87 (54.0)	Reference		
26-35 yr	293 (23.2)	116 (39.6)	177 (60.4)	0.77 (0.52‒1.14)		
36-45 yr	251 (19.9)	129 (51.4)	122 (48.6)	1.24 (0.84‒1.85)		
46-55 yr	179 (14.2)	102 (57.0)	77 (43.0)	1.56 (1.02‒2.39)		
≥56 yr	214 (16.9)	133 (62.1)	81 (37.9)	1.93 (1.28‒2.92)		
**Days from illness onset to diagnosis**						
Median (interquartile range)	3 (2–5)	3 (2–4)	3 (2–5)			
**Symptoms — no./total no. (%)**						
Fever	1150/1264 (91.0)	586/638 (91.8)	564/626 (90.1)	1.24 (0.84‒1.82)	0.277	0.308
Rash	447/1264 (35.4)	275/638 (43.1)	172/626 (27.5)	2.00 (1.58‒2.53)	< 0.001	0.010
Arthralgia	591/1264 (46.8)	370/638 (58.0)	221/626 (35.3)	2.53 (2.02‒3.18)	< 0.001	0.010
Myalgia	720/1264 (57.0)	402/638 (63.0)	318/626 (50.8)	1.65 (1.32‒2.07)	< 0.001	0.010
Conjunctivitis	86/1264 (6.8)	58/638 (9.1)	28/626 (4.5)	2.14 (1.34‒3.40)	0.001	0.010
Headache	556/1264 (44.0)	267/638 (41.8)	289/626 (46.2)	0.84 (0.67‒1.05)	0.112	0.140
Sore throat	101/1264 (8.0)	26/638 (4.1)	75/626 (12.0)	0.31 (0.20‒1.50)	< 0.001	0.010
Fatigue	381/1264 (30.1)	191/638 (29.9)	190/626 (30.4)	0.98 (0.77‒1.25)	0.873	0.873
Vomiting	152/1264 (12.0)	58/638 (9.1)	94/626 (15.0)	0.57 (0.40‒0.80)	0.001	0.010
Diarrhea	71/1264 (5.6)	26/638 (4.1)	45/626 (7.2)	0.55 (0.33‒0.90)	0.018	0.026

FDR: False discovery rate. Statistical significance was set at FDR < 0.05 (symptoms) and *p* < 0.05 (sex and age). Only symptoms include FDR-adjusted *p*-values.

Fever was the most frequently reported symptom among confirmed cases (91.8%), although it was not significantly associated with infection (*p* = 0.277; FDR = 0.308). In contrast, myalgia (63.0%), arthralgia (58.0%), and rash (43.1%) were significantly associated with CHIKV infection (myalgia: odds ratio [OR] 1.65, 95% confidence interval [CI] 1.32–2.07, *p* < 0.001; FDR = 0.010; arthralgia: OR 2.53, 95% CI 2.02–3.18, *p* < 0.001; FDR = 0.010; rash: OR 2.00, 95% CI 1.58–2.53, *p* < 0.001; FDR = 0.010). Conjunctivitis, reported in 58 cases (9.1%), was also significantly associated with infection (OR 2.14, 95% CI 1.34–3.40, *p* = 0.001; FDR = 0.010). Headache and fatigue were observed in 41.8% and 29.9% of CHIKV infection cases, respectively, but neither was significantly associated with the disease (headache: *p* = 0.112; FDR = 0.140; fatigue: *p* = 0.873; FDR = 0.873). Other symptoms, including sore throat (4.1%), vomiting (9.1%), and diarrhea (4.1%), were less frequent and negatively associated with infection (sore throat: OR 0.31, 95% CI 0.20–0.50, *p* < 0.001; FDR = 0.010; vomiting: OR 0.57, 95% CI 0.40–0.80, *p* = 0.001; FDR = 0.010; diarrhea: OR 0.55, 95% CI 0.33–0.90, *p* = 0.018; FDR = 0.026).

We next assessed the association between clinical features and age among CHIKV-infected patients (Table 2). Significant age-dependent variations were observed. Fever was reported in all patients aged ≤ 15 years but declined to 86.8% and 88.2% among those aged 36–45 and 46–55 years, respectively (*p* = 0.005; FDR = 0.0125). The rash was most common in the youngest group (54.8%) and decreased progressively with age, reaching 26.3% among individuals aged ≥ 56 years (*p* < 0.001; FDR = 0.0100). Arthralgia was reported more frequently in older age groups, peaking at 67.6% among individuals aged 46–55 years and 65.9% among those aged 36–45 years, and was least common among patients aged ≤ 15 years (29.8%) (*p* < 0.001; FDR = 0.0100). Myalgia was also associated with age, being most frequent among individuals aged 26–35 years (69.8%) and 36–45 years (68.2%) and less common among those aged ≤ 15 years (44.0%) (*p* = 0.003; FDR = 0.0100). By contrast, the prevalence of conjunctivitis, headache, sore throat, fatigue, vomiting and diarrhea did not vary significantly across age groups.

**Table 2:** Clinical features by age group among CHIKV-infected patients (N = 638). The table shows the proportion of patients in each age group reporting fever, rash, arthralgia, myalgia, vomiting, conjunctivitis, headache, sore throat, fatigue, and diarrhea. *P*-values and FDR-adjusted *p*-values (false discovery rate) are provided for comparisons across age groups. Statistical significance was defined as FDR < 0.05.

Clinical features	CHIKV-infected cases -no. (%)	Age distribution — no. (%)						*p*-value	FDR-adjusted *p*-value
		≤ 15 yr (n = 84)	16‒25 yr (n = 74)	26‒35 yr (n = 116)	36‒45 yr (n = 129)	46‒55 yr (n = 102)	≥ 56 yr (n = 133)		
Fever	586 (91.8)	84 (100.0)	69 (93.2)	111 (95.7)	112 (86.8)	90 (88.2)	120 (90.2)	0.005	**0.0125**
Rash	275 (43.1)	46 (54.8)	37 (50.0)	56 (48.3)	64 (49.6)	37 (36.3)	35 (26.3)	<0.001	**0.0100**
Arthralgia	370 (58.0)	25 (29.8)	42 (56.8)	70 (60.3)	85 (65.9)	69 (67.6)	79 (59.4)	<0.001	**0.0100**
Myalgia	402 (63.0)	37 (44.0)	44 (59.5)	81 (69.8)	88 (68.2)	69 (67.6)	83 (62.4)	0.003	**0.0100**
Conjunctivitis	58 (9.1)	8 (9.5)	4 (5.4)	10 (8.6)	14 (10.9)	10 (9.8)	12 (9.0)	0.874	0.8740
Headache	267 (41.8)	33 (39.3)	41 (55.4)	50 (43.1)	53 (41.4)	35 (34.3)	55 (41.4)	0.140	0.2000
Sore throat	26 (4.1)	6 (7.1)	4 (5.4)	6 (5.2)	2 (2.3)	5 (4.9)	3 (2.3)	0.242	0.3025
Fatigue	191 (29.9)	22 (26.2)	26 (35.1)	42 (36.2)	34 (28.6)	29 (28.4)	38 (28.6)	0.453	0.5033
Vomiting	58 (9.1)	14 (16.7)	8 (10.8)	12 (10.3)	5 (9.0)	7 (6.9)	12 (9.0)	0.048	0.0960
Diarrhea	26 (4.1)	0 (0.0)	3 (4.1)	3 (2.6)	7 (6.0)	5 (4.9)	8 (6.0)	0.191	0.3183

Bold numbers represent statistically significant FDR-adjusted *p*-values (FDR < 0.05).

### CHIKV viral load and clinical symptom associations

**Fig 3:**
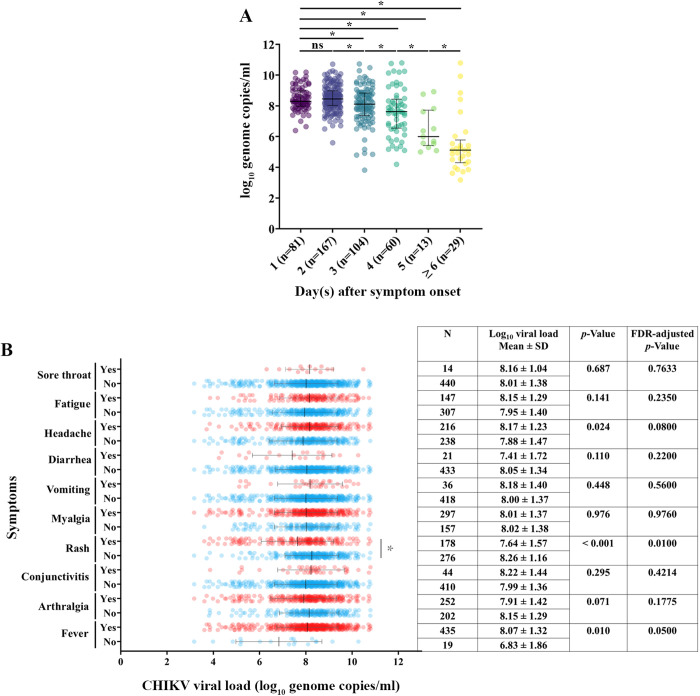
Correlation between CHIKV viral load, timing of symptom onset, and clinical outcomes (N = 454). (A) The correlation between CHIKV viral load and timing of sample collection relative to symptom onset. (B) Analysis of the association between CHIKV viral load and different clinical feature groups. Each data point corresponds to an individual patient. (*; FDR-adjusted *p* value < 0.05).

A total of 454 serum samples positive for CHIKV by RT-PCR with sufficient serum volume were analyzed to quantify the viral RNA copy number. The association between CHIKV log₁₀ viral load and timing of sampling relative to symptom onset was assessed. Mean viral loads were high on day 1 (8.32 log₁₀ copies/mL) and day 2 (8.56 log₁₀ copies/mL), with no significant difference between these time points. Viral loads declined progressively from day 3 onward (Fig 3A). Associations between viral load and clinical symptoms were also examined. The presence of rash was associated with a significantly lower mean viral load compared with the mean viral load in those without rash (7.64 vs 8.26; FDR = 0.010). Patients with fever had higher mean viral loads than those without fever, but this difference was only borderline significant (8.07 vs 6.83; FDR = 0.050). No significant differences in viral load were observed between patients with and without headache, fatigue, diarrhea, arthralgia, myalgia, conjunctivitis, or vomiting (Fig 3B).

We next assessed independent predictors of key clinical manifestations using multivariate logistic regression among 454 CHIKV RT-PCR–confirmed cases. Five symptoms (fever, rash, arthralgia, myalgia, and conjunctivitis) were selected based on significant univariate associations (Table 1) or high prevalence among CHIKV-positive patients (e.g., fever >90%). The model adjusted for age (per 10-year increase), sex, day since illness onset, and CHIKV viral load (log₁₀ copies/mL). As shown in Table 3, rash was independently associated with younger age (adjusted odds ratio [aOR] = 0.73; 95% CI: 0.65–0.83; p < 0.001), female sex (aOR = 1.78; 95% CI: 1.18–2.68; p = 0.006), and lower viral load (aOR = 0.72; 95% CI: 0.60–0.85; p < 0.001). Arthralgia was associated with older age (aOR = 1.13; 95% CI: 1.02–1.26; p = 0.021) and later days after symptom onset (aOR = 1.14; 95% CI: 1.00–1.30; p = 0.045). Myalgia was more frequent among older individuals (aOR = 1.18; 95% CI: 1.06–1.32; p = 0.003), whereas fever was associated with higher viral loads (aOR = 1.54; 95% CI: 1.08–2.19; p = 0.016). No independent predictors were identified for conjunctivitis.

**Table 3:** Multivariate logistic regression identifying independent predictors of key clinical symptoms among RT-PCR–confirmed CHIKV cases (N = 454). For each symptom (fever, rash, arthralgia, myalgia, conjunctivitis), associations with age (per 10-year increase), sex (female vs male), log_10_ CHIKV viral load (copies/mL), and days from illness onset to sample collection were evaluated. Adjusted odds ratios (aORs), 95% confidence intervals (CIs), and p-values are shown.

Symptom	Variable	aOR (95% CI)	*p*-value
**Fever**	Age (per 10 years)	0.88 (0.66–1.18)	0.398
	Sex (female vs. male)	0.61 (0.22–1.68)	0.335
	Viral load (log₁₀ copies/mL)	1.54 (1.08–2.19)	**0.016**
	Day of illness onset	0.96 (0.81–1.14)	0.649
**Rash**	Age (per 10 years)	0.73 (0.65–0.83)	**<0.001**
	Sex (female vs. male)	1.78 (1.18–2.68)	**0.006**
	Viral load (log₁₀ copies/mL)	0.72 (0.60–0.85)	**<0.001**
	Day of illness onset	1.03 (0.92–1.15)	0.619
**Arthralgia**	Age (per 10 years)	1.13 (1.02–1.26)	**0.021**
	Sex (female vs. male)	1.40 (0.96–2.05)	0.084
	Viral load (log₁₀ copies/mL)	0.96 (0.81–1.13)	0.614
	Day of illness onset	1.14 (1.00–1.30)	**0.045**
**Myalgia**	Age (per 10 years)	1.18 (1.06–1.32)	**0.003**
	Sex (female vs. male)	1.04 (0.70–1.54)	0.863
	Viral load (log₁₀ copies/mL)	0.98 (0.83–1.16)	0.851
	Day of illness onset	0.98 (0.88–1.09)	0.720
**Conjunctivitis**	Age (per 10 years)	1.05 (0.89–1.25)	0.555
	Sex (female vs. male)	1.72 (0.89–3.34)	0.106
	Viral load (log₁₀ copies/mL)	1.12 (0.85–1.47)	0.432
	Day of illness onset	0.97 (0.79–1.18)	0.741

Bold values indicate statistical significance at *p* < 0.05.

### Phylogenetic and nucleotide identity analysis of global chikungunya virus sequences in comparison with Thai strains (2020–2023)

**Fig 4:**
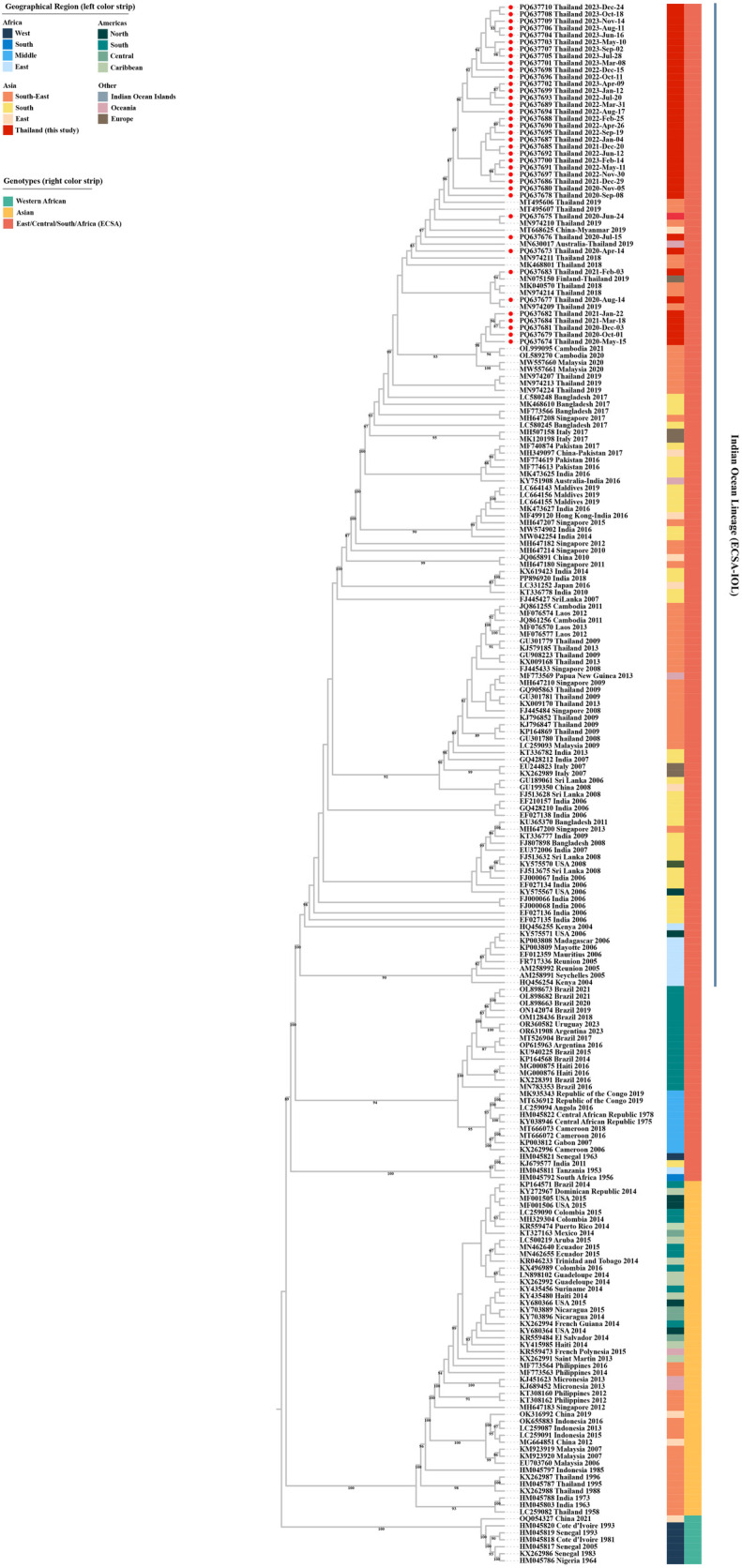
Phylogenetic tree analysis of CHIKV complete coding sequences. A maximum-likelihood (ML) phylogenetic tree was constructed using the complete coding sequences of CHIKV strains from Thailand identified in this study, along with various strains obtained from the GenBank database. The tree was inferred using the GTR + F + I + G4 substitution model with 1000 ultrafast bootstrap replicates. Bootstrap values ≥80 are shown at major nodes. Red dots indicate CHIKV strains from Thailand identified in this study (GenBank accession numbers PQ637673–PQ637710). The left color strip represents the geographic regions of the isolates, and the right color strip indicates the viral genotypes.

Phylogenetic and nucleotide identity analyses were conducted on 38 CHIKV isolates from Thailand (GenBank accession numbers PQ637673–PQ637710) collected between 2020 and 2023. The complete coding sequences (11,238 nucleotides) of these isolates were compared with 186 global isolates retrieved from GenBank, representing virus discoveries from 1953 to 2021 (Fig 4 and S2 table).

Maximum-likelihood phylogenetic analysis showed that CHIKV strains obtained in this study from cases in Thailand (2020–2023) clustered within the Indian Ocean lineage (ECSA-IOL), a distinct epidemic clade of the East/Central/South African (ECSA) genotype. These strains shared 99.6–99.9% nucleotide identity with Thai strains from 2018–2019, 99.5–99.8% with isolates from Malaysia (2020) and Cambodia (2020–2021), and 99.6–99.9% with a strain from China (2019) isolated from a traveler returning from Myanmar. In contrast, the newly sequenced strains were genetically distinct from CHIKV strains circulating in Thailand during 2008–2013 and strains reported in Singapore, Cambodia, Laos, and Malaysia during the same period. Comparative analysis of the complete coding region revealed 98.7–99.0% nucleotide identity with Thai strains from 2008–2013 and 98.6–99.0% identity with Southeast Asian strains from the same period.

Additionally, Thai CHIKV strains from 2020–2023 revealed 98.6–99.8% nucleotide similarity with global viruses within the ECSA-IOL lineage circulating outside Southeast Asia. In contrast, Thai strains of the Asian genotype, which predominated before the emergence of ECSA-IOL, shared only 93.5–94.7% nucleotide identity with the newly sequenced strains. Comparisons of the 2020–2023 Thai strains with other genotypes revealed 92.8–94.5% similarity with the Asian genotype and 96.4–97.6% with ECSA non-IOL lineages. The Western African genotype showed the lowest similarity (84.5–85.0%), indicating substantial inter-genotypic divergence.

### Assessment of the evolutionary history of CHIKV ECSA-IOL

**Fig 5:**
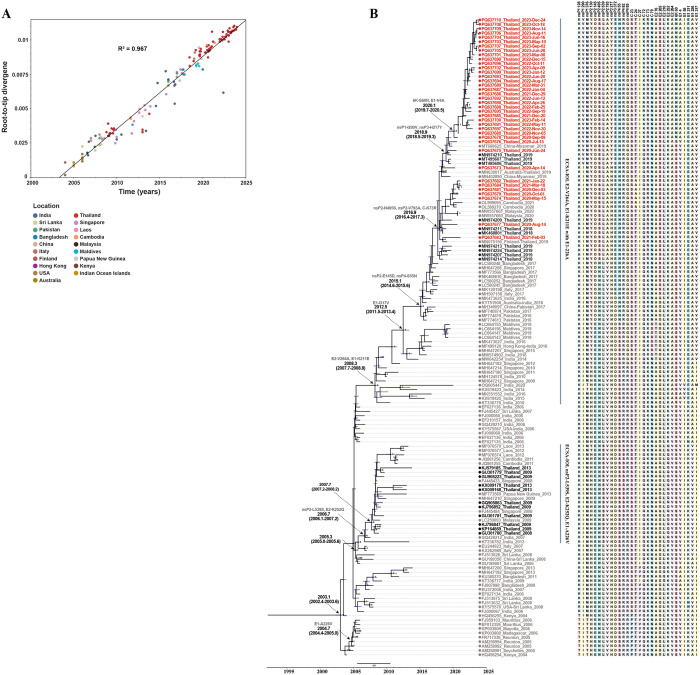
Molecular clock analysis of CHIKV ECSA-IOL. (A) Temporal signal analysis showing the correlation between sequence sampling dates and their genetic distances from the root of the maximum likelihood phylogeny (R² = 0.967). (B) Maximum clade credibility (MCC) tree of the CHIKV ECSA-IOL genotype. Arrows indicate the time to the most recent common ancestor (tMRCA) with corresponding 95% highest posterior density (HPD) intervals and amino acid substitutions. Black nodes represent branches with posterior probability (PP) > 0.95, and blue node bars denote the 95% HPD intervals for node heights. Sequences from Thailand identified in this study (GenBank accession numbers PQ637673–PQ637710) are labeled in red. Previously published Thai strains are shown in black, and other global reference sequences are colored grey. Sequences are named following the format: accession number_country_collection year. Amino acid mutations specific to each isolate are shown next to the sequence tips in the MCC tree.

To determine the evolutionary dynamics of recent CHIKV ECSA-IOL strains in Thailand and their genetic relationships with earlier Thai isolates and global ECSA-IOL viruses, a dataset comprising 147 full-length coding sequences was compiled. This dataset included strains from Kenya (2004), Africa, the Indian Ocean islands, the Indian subcontinent, Europe, East Asia, and Southeast Asia, up to the most recent outbreaks in 2023. A maximum-likelihood phylogenetic tree was generated to perform root-to-tip divergence regression analysis. The root-to-tip plot demonstrated a strong clock-like signal (R² = 0.967; correlation coefficient = 0.983), confirming the suitability of the dataset for molecular clock analysis (Fig 5A). Time-scaled maximum clade credibility (MCC) phylogeny was then constructed using an uncorrelated lognormal relaxed clock model and a Bayesian skyline coalescent prior (Fig 5B). The analysis estimated that the most recent common ancestor (tMRCA) of CHIKV ECSA-IOL arose in February 2003, with a 95% highest posterior density (HPD) interval ranging from May 2002 to August 2003. The estimated evolutionary rate was 5.03 × 10 ⁻ ⁴ substitutions per site per year (95% HPD: 4.58 × 10 ⁻ ⁴–5.50 × 10 ⁻ ⁴). Molecular clock analysis suggested that ECSA-IOL viruses have circulated in mainland Southeast Asia since September 2007 (95% HPD March 2007–March 2008), following the Indian Ocean outbreaks.

The initial expansion of ECSA-IOL viruses in mainland Southeast Asian countries, including Thailand, involved strains predominantly carrying the E1-A226V substitution. The tMRCA of CHIKV strains carrying E1-A226V was estimated to be August 2004 (95% HPD May 2004–January 2005), coinciding with outbreaks in Réunion Island and the Indian Ocean region. In addition to E1-A226V, strains circulating between 2008 and 2013 commonly harboured nsP2-L539S and E2-K252Q mutations, observed in Thailand, Malaysia (2009), Singapore (2008–2009), Cambodia (2011), and Laos (2012–2013). These strains were estimated to have originated in India around September 2006 (95% HPD February 2006–March 2007). In contrast, the CHIKV strains identified in this study did not carry the E1-A226V, nsP2-L539S, or E2-K252Q mutations. Instead, they exhibited two amino acid substitutions—E2-V264A and E1-K211E—and retained alanine at position E1-226 (E1-226A). These substitutions were shared with strains reported from Thailand (2018–2019), Cambodia (2020–2021), Myanmar (2019), Malaysia (2020), Singapore (2009–2012, 2015, 2017), India (2010, 2014–2016, 2020), Pakistan (2016–2017), Bangladesh (2017), the Maldives (2019), and Italy (2017). Phylogenetic analysis demonstrated that these viruses formed distinct monophyletic clades, separate from earlier ECSA-IOL strains harboring E1-A226V. The tMRCA of CHIKV strains harboring E2-V264A, E1-K211E, and E1-226A was estimated to be April 2008, with a 95% HPD between August 2007 and October 2008 and a posterior probability (PP) of 1. The analysis estimated that ECSA-IOL strains carrying these mutations emerged in Thailand in November 2016 (95% HPD May 2016–April 2017).

Several amino acid substitutions distinguishing the ECSA-IOL strains identified in this study from Thai CHIKV strains detected between 2008 and 2013 were observed. All strains harbored E1-I317V, nsP2-E145D, nsP2-N495S, nsP4-S55N, and C-K73R substitutions. Among them, 29 strains (PQ637676, PQ637678, PQ637680, PQ637685–PQ637710) exhibited E1-V4A and 6K-S60N mutations. Additionally, 31 strains collected between 2020 and 2023 shared nsP1-I290V and nsP3-H217Y substitutions with Thai strains from 2019 and a strain isolated in China in 2019 from a traveler returning from Myanmar. Among these, and based on Thai ECSA-IOL sequences generated in this study and included in the time-scaled phylogenetic analysis, the frequencies of E1-V4A and 6K-S60N, as well as nsP1-I290V and nsP3-H217Y, increased from <50% in 2020–2021 to 100% in 2022–2023 (Fig 5), suggesting persistence of these variants in recent years.

## Discussion

This study characterises the epidemiology, laboratory diagnostic approaches, demographic distribution, and clinical manifestations of CHIKV infection during and after the COVID-19 pandemic in Thailand, and provides a comprehensive analysis of the genetic characteristics and evolutionary dynamics of circulating strains. National surveillance and our study surveillance showed a marked decline in CHIKV transmission during 2021, likely resulting from a combination of factors, including a true reduction in virus circulation due to strict COVID-19-related mobility restrictions that probably reduced human–vector contact, and disruptions in healthcare access, diagnostic capacity, and surveillance systems. The redirection of public health resources toward the COVID-19 response may have further contributed to underreporting, particularly of mild or undocumented CHIKV infections. The resurgence of cases in 2022–2023, following the easing of movement restrictions, highlights the persistent endemicity of CHIKV in Thailand and underscores the need for ongoing molecular surveillance and strengthened, integrated vector control efforts to mitigate future outbreaks.

Individuals infected with CHIKV exhibit a broad range of clinical manifestations that overlap with other arboviral diseases, rendering laboratory testing essential for accurate diagnosis [46,47]. Our findings confirm that RT-PCR remains the gold standard for early CHIKV detection, with positivity exceeding 90% within the first four days, aligning with the peak viraemia observed in this and previous studies [48,49]. CHIKV IgM levels remained low initially but increased from day 5 onward, surpassing RT-PCR after day 6, thus rendering serology more effective for later-stage diagnosis. The delayed IgG response further underscores its role in identifying past infections. These findings support a stage-specific diagnostic approach combining molecular and serological testing. Incorporating this approach into national diagnostic algorithms for acute febrile illness could facilitate timely differentiation of CHIKV from other arboviral infections, such as dengue and Zika. Specifically, RT-PCR should be prioritized during days 1–4 of illness, followed by IgM detection from day 5 onward, to optimize diagnostic accuracy throughout the disease course. Such integration would reduce misdiagnosis, accelerate case confirmation, and improve outbreak response efficiency.

We found that CHIKV infection affected individuals across all age groups; however, age-specific differences indicated that older adults, particularly those aged ≥56 years, had the highest infection prevalence, followed by those aged 46–55 years. Previous studies have demonstrated that the severity and chronicity of chikungunya disease increase with age [50–52], while current evidence links comorbidities such as hypertension, obesity, diabetes, and cardiovascular disease to a heightened risk of severe outcomes [53]. Therefore, older adults, particularly those with underlying conditions, should be recognized as a high-risk group and encouraged to monitor for severe symptoms during outbreaks. Nevertheless, severe complications, including myocarditis, encephalopathy, and multiorgan failure, were not observed in this cohort. As previously reported [54–57], our study demonstrates a broad spectrum of clinical features in CHIKV-infected individuals, including fever, myalgia, arthralgia, rash, and headache. Remarkably, we found that myalgia, arthralgia, rash, and conjunctivitis were significantly associated with CHIKV infection, consistent with previous reports identifying musculoskeletal symptoms as key features [58] Although less frequent, the significant association of conjunctivitis with CHIKV supports previous findings that it occurs more commonly in CHIKV cases than in dengue or other febrile illnesses [58]. Age-specific differences in clinical presentation were evident in our cohort. Rash was significantly more common among younger patients (≤15 years) and decreased progressively with age, a finding consistent with previous studies noting a higher incidence of skin rash in children [59,60]. Our study also observed that arthralgia was significantly less common in children, supporting previous evidence that CHIKV infections in children are characterized more by rash than by joint pain [59]. The observed age-related differences in joint symptoms are consistent with previous outbreaks, such as in Kerala, where joint pain was more frequently reported as an initial symptom among older adults [61]. These findings further support existing evidence that increasing age is an independent risk factor for the development of persistent musculoskeletal disorders or rheumatic complications following chikungunya infection [3,62–64]

We examined the association between CHIKV viral load and time from symptom onset and found that RNA copy numbers peaked within the first two days before declining. These findings are consistent with previous reports showing high viremia early in the illness, followed by decreases as CHIKV-specific antibody responses develop and contribute to viral clearance [65,66]. It is important to acknowledge that this analysis was conducted using a single serum sample per participant, rather than longitudinal sampling. Consequently, interindividual variability and the lack of serial data may limit our ability to characterize intra-host viral kinetics comprehensively. Nonetheless, the observed trend is consistent with established patterns of CHIKV infection dynamics. Although the data indicate a general decline in CHIKV viraemia over time, substantial interindividual variability was observed, likely driven by host-specific immune responses, viral factors, and genetic susceptibility [67–69].

We further explored the associations between CHIKV viral load and clinical manifestations. Our findings demonstrate that, after correcting for multiple comparisons, only the presence of rash was significantly associated with viral load among individuals infected with CHIKV. Patients with rash exhibited significantly lower mean viral loads compared with those without rash (7.64 vs 8.26; FDR = 0.010), which is consistent with reports from India that show higher viremia in individuals without rash [70]. The association between fever and higher viral loads in our cohort was only borderline significant after adjustment (8.07 vs 6.83; FDR = 0.050), whereas a previous study from Thailand identified a statistically significant relationship between fever and viral load [71]. In contrast to that study, we did not observe a significant association between headache and viral load after correcting for multiple comparisons, despite an uncorrected p-value of 0.024. Similarly, although Silva et al. [64] reported lower viral loads in younger patients with myalgia, this correlation was not observed in our cohort. These differences may reflect variations in study design, population characteristics, or host immune responses.

To determine whether the associations observed in the univariate analyses persisted after adjusting for other variables, we performed a multivariate logistic regression including age, sex, day since illness onset, and viral load. In the univariate analysis, viral load was higher in patients with fever than in those without, but the difference was only borderline significant; in contrast, the multivariate model, treating viral load as a continuous variable, identified a significant association, suggesting that systemic febrile responses may be partly driven by the extent of viral replication. Rash maintained its inverse relationship with viral load and was also linked to younger age and female sex. Arthralgia remained associated with older age, with later illness onset emerging as an additional factor, and myalgia continued to be more common in older individuals. These findings highlight the interplay between host characteristics, viral replication, and symptom expression in acute CHIKV infection.

Our viral genome analysis revealed that CHIKV strains circulating in Thailand between 2020 and 2023 belonged to the ECSA-IOL genotype, consistent with previous reports describing the predominance of this genotype in Thailand and neighboring Southeast Asian countries since its introduction [20,72–74]. However, despite sharing the same genotype, the 2020–2023 Thai strains formed distinct clades, separate from historical isolates identified between 2008 and 2013. The CHIKV Thai strains circulating between 2008 and 2013 harbored the E1-A226V substitution alongside the nsP2-L539S and E2-K252Q mutations. The E1-A226V substitution has been shown in laboratory studies to enhance CHIKV infectivity in *Aedes albopictus* by increasing midgut infectivity, promoting viral dissemination to the salivary glands, and facilitating transmission to vertebrate hosts [75]. The co-occurrence of nsP2-L539S and E2-K252Q has also been experimentally associated with enhanced CHIKV fitness in *Aedes albopictus* [76]. These mutations have previously been associated with CHIKV outbreaks in regions where *Aedes albopictus* was reported to be prevalent, such as rubber plantation areas in southern Thailand during 2008–2009 and in Bueng Kan Province in 2013, based on studies conducted during those outbreaks [19–21,77]. However, our study did not assess mosquito populations in the sampled areas, and no entomological data were collected.

By contrast, CHIKV strains detected between 2020 and 2023 lacked mutations previously associated with *Aedes albopictus* adaptation (E1-A226V, nsP2-L539S, and E2-K252Q) [75,76] and instead harbored E1-K211E and E2-V264A substitutions on an E1-226A background.

Viruses carrying these substitutions have demonstrated enhanced fitness for *Aedes aegypti* without increased adaptation to *Aedes albopictus* [78]. Molecular clock analysis suggests that this double-mutant virus likely emerged around 2008 in India. These ECSA-IOL mutations were initially detected in CHIKV isolates from southern India between 2009 and 2010 [79], subsequently identified in isolates from New Delhi in late 2010 [80], and later observed in *Aedes aegypti*–dominated areas of Kolkata during 2011 and 2012 [81]. Since then, the virus harboring mutations previously associated with increased fitness in *Aedes aegypti* [78] has spread across the Indian subcontinent, leading to outbreaks in Pakistan (2016–2017) and Bangladesh (2017) [82–85]. Our findings suggest that CHIKV strains detected in Thailand since 2018 did not arise through adaptive genetic changes from the ECSA-IOL viruses responsible for the 2008–2009 outbreaks. Instead, CHIKV strains harboring the E1-K211E and E2-V264A substitutions were likely introduced from the Indian subcontinent around 2016–2017, leading to a large outbreak from late 2018–2019 and sustaining transmission to the present.

Notably, in contrast to the 2008–2013 outbreaks that occurred predominantly in rural areas [20,21], recent CHIKV strains harboring the E1-K211E and E2-V264A substitutions on an E1-226A background have been detected in urban settings in Thailand, where *Aedes aegypti* is known to be prevalent, increasing transmission risks in densely populated areas [19]. Although these mutations have been experimentally associated with increased CHIKV fitness in *Aedes aegypti* [76], no entomological surveillance was conducted in our study areas. Therefore, while our genomic data suggest possible vector-specific adaptation, definitive conclusions regarding vector adaptation in the present outbreak cannot be drawn without supporting field data. Nevertheless, based on known national vector ecology, in urban areas where *Aedes aegypti* is known to predominate, intensified source reduction and community campaigns are recommended, whereas rural areas with documented *Aedes albopictus* activity may require tailored approaches. Implementing such area-specific vector control strategies, in combination with genomic and entomological surveillance, could improve the effectiveness of outbreak prevention and control efforts.

Our findings further indicate that CHIKV strains circulating in Thailand between 2018 and 2023 share high genetic similarity with strains responsible for outbreaks in neighboring countries, including Cambodia (2020–2021) and Malaysia (2020). The genetic similarities, along with the presence of shared amino acid substitutions, including nsP2-N495S, nsP2-V793A, and C-K73R, suggest potential cross-border transmission and regional dispersal of this CHIKV variant. These findings highlight the need for strengthened regional surveillance to track viral movement and mitigate future outbreaks. The close genetic relatedness between Thai strains and those from other Southeast Asian countries highlights the potential for cross-border transmission. High regional connectivity, including frequent population movements across porous borders, likely accelerates viral spread. Coordinated regional surveillance of travel-associated cases, combined with integrated genomic datasets from multiple countries, is essential for mapping transmission routes and implementing targeted vector control. Establishing a coordinated national surveillance system is therefore critical to guide targeted interventions, particularly in differentiating outbreak foci, prioritizing high-risk areas, and integrating entomological and genomic monitoring into routine public health practice. Integrating clinical and genomic surveillance into national and regional reporting systems would strengthen early outbreak detection and situational awareness, particularly in border areas with high population movement. Such an approach would enable targeted vector control, enhance regional preparedness, and facilitate coordinated responses across Southeast Asia, reducing the risk of large-scale CHIKV outbreaks.

Additionally, the CHIKV genomes analyzed in this study harbored multiple nonsynonymous mutations. Notably, the frequencies of E1-V4A and 6K-S60N, as well as nsP1-I290V and nsP3-H217Y, increased from <50% in 2020–2021 to 100% in 2022–2023, indicating persistence and regional spread of this variant. While such increases may reflect ongoing adaptation, formal selection analyses were not performed and these trends are interpreted cautiously. However, the functional significance of these mutations remains unclear, and further studies are needed to assess their effects on viral replication, immune evasion, and transmission dynamics to inform risk assessment and control strategies.

This study has several limitations. First, only approximately 7% (38/524) of RT-PCR–confirmed CHIKV cases underwent genome sequencing, which may introduce selection bias and limit the representativeness of the observed genetic diversity. Second, data on patient comorbidities (e.g., diabetes, hypertension, immunosuppression) were not collected as part of the study protocol and could not be accessed from referring hospitals. Our data collection focused primarily on clinical symptoms at presentation, which may limit the ability to assess the impact of underlying health conditions on disease severity. Future studies should incorporate systematic comorbidity assessment to improve risk stratification and better elucidate age-related differences in clinical presentation. Third, although all samples were also tested for DENV and ZIKV RNA, the typically short duration of viremia for these infections may limit RNA detection. In addition, serological testing for DENV and ZIKV was not performed; therefore, co-infections cannot be completely excluded. Fourth, while all enrolled patients were residents of Thailand and none reported recent travel outside their province or abroad, travel history was not available for every case, raising the possibility of misclassification between imported and autochthonous infections. Finally, all clinical and viral load data were obtained from a single serum sample per patient, without longitudinal follow-up, which limits our ability to assess within-host viral kinetics or the temporal progression of symptoms.

In summary, this study integrates epidemiological, clinical, viral load, and genomic data to provide a comprehensive characterization of CHIKV circulation in Thailand during 2020–2023. We identified associations between clinical manifestations and patient sex, age, illness duration, and viral load, revealing distinct age-related symptom patterns and links between viral load and specific clinical features. Our findings support stage-specific diagnostic algorithms, document the persistence and regional spread of ECSA-IOL variants, and highlight the need for sustained molecular surveillance integrated with future entomological and epidemiological data to detect emerging variants, guide targeted vector control, and strengthen outbreak preparedness in Thailand and across Southeast Asia.

## Supporting information

S1 File BEAST XML input files for Bayesian time-scaled phylogenetic analyses. Contains XML configuration files for four independent MCMC runs (150 million generations each) used for ECSA-IOL CHIKV phylogenetic reconstruction.
(ZIP)

S2 File MCMC log outputs from BEAST runs 1 and 2. Includes log files generated from the first two independent MCMC runs.
(ZIP)

S3 File MCMC log outputs from BEAST runs 3 and 4. Includes log files generated from the remaining two independent MCMC runs.
(ZIP)

S1 Fig Flow diagram of patient enrollment, laboratory testing, sample selection for genome sequencing, CHIKV viral load quantification, and phylogenetic analyses. Serum samples from 1,264 suspected cases were tested for CHIKV, dengue virus (DENV), and Zika virus (ZIKV) by RT-PCR, and for CHIKV-specific IgM and IgG by fluorescence immunoassay. No CHIKV-positive cases had DENV or ZIKV co-infection. Of 524 RT-PCR–positive CHIKV cases, 454 had sufficient serum for viral load quantification. Thirty-eight samples with low Ct values (<30) and broad geographic and temporal representation were selected for complete coding genome sequencing and analyzed by maximum-likelihood (38 Thai sequences from this study + 186 global sequences from GenBank) and Bayesian time-scaled phylogenetics (38 Thai sequences from this study + 109 ECSA-IOL sequences).
(TIF)

S2 Fig Standard curve used for CHIKV viral load quantification by qRT-PCR. Tenfold serial dilutions of in vitro–transcribed CHIKV RNA (10¹–10^10^ copies) were used to construct the qRT-PCR standard curve. The curve yielded a slope of –3.402, a Y-intercept of 39.83, a coefficient of determination (R^2^) of 0.997, and an amplification efficiency of 96.77%. This standard curve was used to determine CHIKV RNA copy numbers in patient serum samples.
(TIF)

S1 Table Primers used for amplification of the complete coding region of the chikungunya virus genome. Four pairs of primers were used to generate overlapping RT-PCR products spanning the complete coding region of the CHIKV genome.
(DOCX)

S2 Table Percentage of nucleotide identity among CHIKV strains. The table shows the pairwise comparison (%) of nucleotide identity based on complete genome sequences for 224 representative CHIKV strains.
(XLSX)
