# Supplementary figures and images for: Chikungunya virus in Thailand (2020–2023): Epidemiology, clinical features, and genomic insights
Source: PLoS Negl Trop Dis. 2025 Sep 15;19(9):e0013548. doi: 10.1371/journal.pntd.0013548 (PMC12449006; doi:10.1371/journal.pntd.0013548)

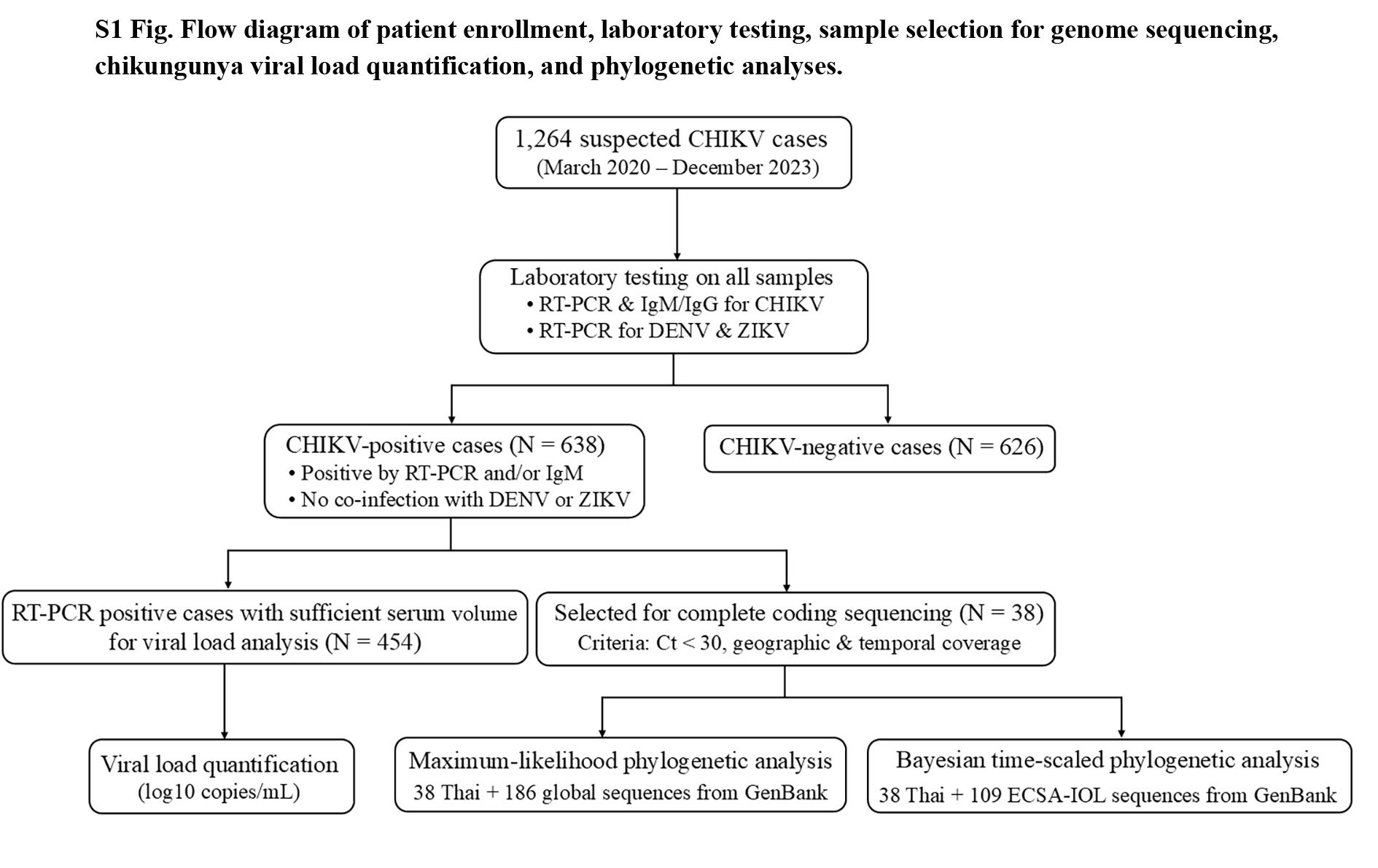

Supplement: S1 Fig — Serum samples from 1,264 suspected cases were tested for CHIKV, dengue virus (DENV), and Zika virus (ZIKV) by RT-PCR, and for CHIKV-specific IgM and IgG by fluorescence immunoassay. No CHIKV-positive cases had DENV or ZIKV co-infection. Of 524 RT-PCR–positive CHIKV cases, 454 had sufficient serum for viral load quantification. Thirty-eight samples with low Ct values (<30) and broad geographic and temporal representation were selected for complete coding genome sequencing and analyzed by maximum-likelihood (38 Thai sequences from this study + 186 global sequences from GenBank) and Bayesian time-scaled phylogenetics (38 Thai sequences from this study + 109 ECSA-IOL sequences). (TIF) [file pntd.0013548.s004.tif]

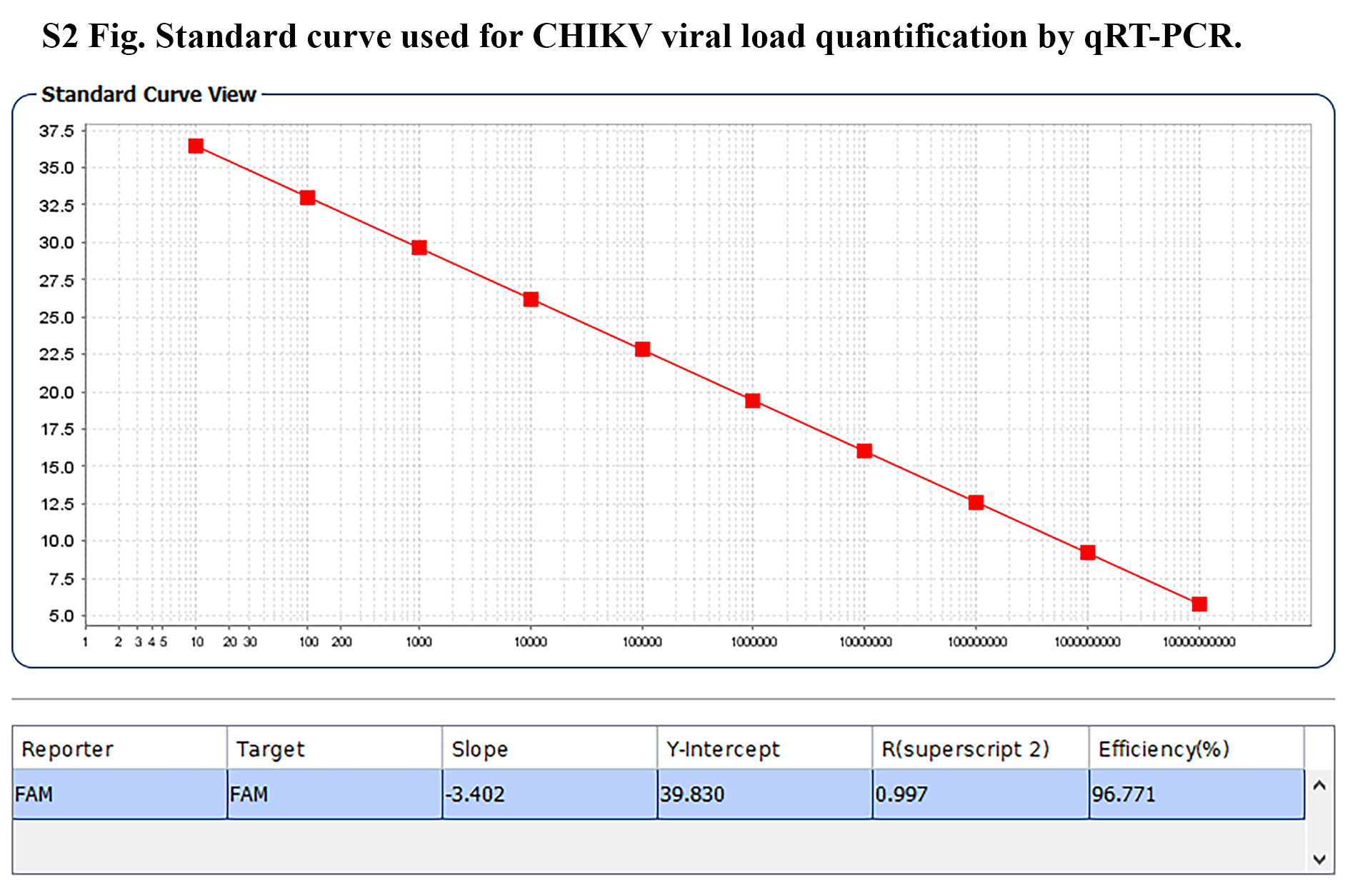

Supplement: S2 Fig — Tenfold serial dilutions of in vitro–transcribed CHIKV RNA (10¹–1010 copies) were used to construct the qRT-PCR standard curve. The curve yielded a slope of –3.402, a Y-intercept of 39.83, a coefficient of determination (R2) of 0.997, and an amplification efficiency of 96.77%. This standard curve was used to determine CHIKV RNA copy numbers in patient serum samples. (TIF) [file pntd.0013548.s005.tif]
