# Supplementary material for: Chikungunya virus in Thailand (2020–2023): Epidemiology, clinical features, and genomic insights
Source: PLoS Negl Trop Dis. 2025 Sep 15;19(9):e0013548. doi: 10.1371/journal.pntd.0013548 (PMC12449006; doi:10.1371/journal.pntd.0013548)
Supplement: S1 Table — Four pairs of primers were used to generate overlapping RT-PCR products spanning the complete coding region of the CHIKV genome. (DOCX) [file pntd.0013548.s006.docx]

**S1 Table. Primers used to obtain the complete coding sequence of CHIKV.** Four pairs of primers were used to generate overlapping RT-PCR products spanning the complete coding region of the CHIKV genome.

| Primer | Nucleotide position | Sequence (5’ to 3’) | Genome region |
| --- | --- | --- | --- |
| Primer 1 Forward | 18 | CACGTAGCCTACCAGTTTCTTA | 5’UTR |
| Primer 1 Reverse | 3504 | GTCTCCTGTTGGCCGGTATAAT | nsP2 |
| Primer 2 Forward | 3332 | TAATAGGCCTGGAGGGAAGATG | nsP2 |
| Primer 2 Reverse | 6380 | CCCAGTATTCTTGGTTGCATG | nsP4 |
| Primer 3 Forward | 6184 | AAAACAGCACGCTTACCACG | nsP4 |
| Primer 3 Reverse | 9861 | AAAGGTTGCTGCTCGTTCCAC | 6K |
| Primer 4 Forward | 9648 | AGTTGTGTCAGTGGCCTCGTTC | E2 |
| Primer 4 Reverse* | 11785 | GTTCGGAGAATCGTGGAAGAG | 3’UTR |

Primers previously published [23] were used; newly designed primers are indicated with an asterisk (*). Nucleotide positions correspond to the CHIKV prototype S27 strain (GenBank accession no. AF369024).
